# Neighborhood disadvantage and multiple myeloma incidence in the Black Women’s Health Study

**DOI:** 10.1093/ije/dyaf188

**Published:** 2025-11-07

**Authors:** Etienne X Holder, Raphael Szalat, Julie R Palmer, Kimberly A Bertrand

**Affiliations:** Slone Epidemiology Center at Boston University, Boston, MA, United States; Boston University Chobanian & Avedisian School of Medicine, Boston, MA, United States; Slone Epidemiology Center at Boston University, Boston, MA, United States; Boston University Chobanian & Avedisian School of Medicine, Boston, MA, United States; Slone Epidemiology Center at Boston University, Boston, MA, United States; Boston University Chobanian & Avedisian School of Medicine, Boston, MA, United States

**Keywords:** multiple myeloma, Black women, neighborhood socioeconomic status, neighborhood disadvantage, women’s health, racial disparity

## Abstract

**Background:**

Black Americans have a >2-fold increased risk of multiple myeloma (MM) compared with other racial/ethnic groups. The underlying biological and environmental mechanisms contributing to this disparity remain poorly understood. Emerging evidence suggests that social and economic factors associated with neighborhoods may influence risk.

**Methods:**

We evaluated associations between two neighborhood-level indices by measuring deprivation and socioeconomic status and MM within the Black Women’s Health Study—a prospective cohort of 59 000 Black women established in 1995. Participant addresses at baseline and over follow-up were geocoded and linked with United States Census Bureau data. We used Cox proportional hazards regression to calculate hazard ratios (HRs) and 95% confidence intervals (CIs) for the association of neighborhood indices, categorized into quartiles, and MM incidence. Multivariable models were adjusted for age, body mass index, educational attainment, alcohol consumption, physical activity, cigarette smoking, and geographic region.

**Results:**

During follow-up through 2021, we identified 276 incident MM cases. In time-varying age-adjusted models, women living in areas of high disadvantage (vs lowest) and low socioeconomic status (vs highest) had a higher incidence of MM than women living in areas of privilege (multivariable HR: 1.48, 95% CI: 1.02, 2.13; HR: 1.28, 95% CI: 0.87, 1.90, respectively).

**Conclusion:**

Black women living in neighborhoods of high concentrated disadvantage or low socioeconomic status have an increased MM risk. Future studies to identify specific neighborhood-level factors that might influence the risk of MM would be beneficial. Ultimately, community reinvestment may offer an opportunity to reduce the increasing burden of MM among Black women.

Key Messages

- We evaluated the associations between social and economic attributes of residential neighborhoods and multiple myeloma (MM) incidence among Black women living in the USA.
- Black American women living in areas of concentrated disadvantage and low socioeconomic status had an increased risk of MM compared with those who lived in more affluent areas, regardless of individual educational attainment.
- Community reinvestment may offer an opportunity to reduce the increasing burden of MM among Black women.

## Background

Multiple myeloma (MM) is the second-most common hematologic malignancy across all racial and ethnic groups, and the most common hematologic malignancy among Black individuals [1, 2]. MM can cause severe complications, including anemia, bone disease, and kidney dysfunction, and, despite the advent of novel therapies, the disease remains incurable, with a 5-year survival rate of 62.4% [2, 3]. The American Cancer Society projects that, in 2025, an estimated 16 080 women will be diagnosed with MM [4]. Black women living in the USA experience a greater burden of disease compared with non-Hispanic White women and were diagnosed with MM at a rate of 14.5 (vs 5.4 among non-Hispanic White women) per 100 000 women in 2021, with the incidence steadily increasing by 1.6% per year [95% confidence interval (CI): 1.3, 2.0] between 2000 and 2021 [5]. Although factors underlying the differences in MM incidence by race are not fully understood, disparities do not appear to be explained by genetic ancestry [6]. Instead, differences by race may be influenced by a combination of social and neighborhood-level factors, including environmental exposures and socioeconomic conditions.

Obesity [7] and pesticides (usually via occupational exposure) [8–11] are the most consistent established modifiable risk factors for MM. Several studies support an association between living in areas of deprivation and cancer incidence [12–16]; however, few have examined this association with MM. Thus, factors associated with the neighborhoods in which Black women live and work may be one area to consider to address the high MM incidence and reduce the burden of disease for Black women. Living in areas of disadvantage puts people at a higher risk of being exposed to obesogenic environments. For example, evidence to date directly links neighborhood disadvantage with chronic stress and inflammation [17–20], which could have a direct impact on cancer development. In addition, residents of lower-resourced neighborhoods may experience limited access to high-quality preventive care [21] and barriers to safe recreation [22].

Two studies based in New York City reported inverse associations between neighborhood walkability—which is associated with increased levels of physical activity and lower rates of obesity [23, 24]—and MM incidence; however, one study was limited to New York City [25] and one was cross-sectional and did not include individual-level data [26]. Finally, in several studies, individual markers of lower socioeconomic status (SES), including lower educational attainment and lower income, have been associated with an increased risk of MM [27–31]; however, no prior studies have considered the potential influence of the socioeconomic context of residential neighborhoods with respect to MM risk.

To address this gap in the literature, we aimed to evaluate the possible relationship between area-level attributes and MM incidence in a large, prospective cohort of Black women.

## Methods

### Study population and design

The women included in this study were Black Women’s Health Study (BWHS) participants. The BWHS is a long-standing, longitudinal cohort of 59 000 self-identified Black women. Participants were aged 21–69 years at enrollment in 1995 and recruited from across the USA via *Essence* magazine subscriber lists and professional organization membership lists [32]. Participants provided information on medical history, demographics, lifestyle factors, and MM risk factors on self-administered questionnaires at baseline and every 2 years. We followed the Strengthening the Reporting of Observational Studies in Epidemiology reporting guidelines.

### Neighborhood indices

We used two neighborhood indices to quantify area-level deprivation: neighborhood-concentrated deprivation (nDIS) [33] and neighborhood SES (nSES) [34]. The development of these indices for the BWHS was previously described [16, 34]. In brief, participant residential addresses at baseline and throughout follow-up were geocoded and linked to US Census data and the American Community Survey data at the block-group level. The nDIS index included the following domains: the percentage of individuals below the poverty line, on public assistance, in female-headed households, unemployed, <18 years of age, and Black residents. In contrast, the nSES index encompassed median household income; median housing value; the percentage of households receiving interest, dividend, or net rental income; the percentage of adults aged ≥25 years with a college degree; the percentage of employed persons aged ≥16 years; and the percentage of two-parent households. The domains for each index were weighted to obtain a single nDIS or nSES score. Higher nDIS scores indicate residence in areas of concentrated disadvantage (vs concentrated privilege). Participants with higher nSES scores lived in more affluent neighborhoods (vs economically deprived). Quartile cut points for nDIS and nSES were based on the distributions of these variables in the entire BWHS population for each questionnaire cycle.

### MM ascertainment

We identified incident cases of MM through self-reports on biennial questionnaires, linkages with state cancer registries in states in which >95% of the BWHS participants live, and linkages with the National Death Index [35]. Self-reported MM diagnoses were confirmed by a review of medical records, pathology reports, and cancer registry records. We did not include smoldering MM in our definition of incident MM.

### Covariate data

Covariates of interest were selected based on the prior literature. They included established or suspected risk factors for MM: weight, height, educational attainment, cigarette-smoking history, alcohol consumption, physical activity, and region of residence. Except for height, all data were updated on subsequent questionnaires. Body mass index (BMI) was calculated as weight in kilograms divided by height in meters squared; the cumulative average BMI was calculated for each follow-up cycle as the time-varying BMI.

### Statistical analysis

Participants were included if their address was able to be geocoded and they had no prior cancer diagnoses (except non-melanoma skin cancer) at baseline (*N* = 52 272). Participants were followed from baseline (1995) until diagnosis of MM or end of follow-up in 2021, whichever occurred first. We modeled nDIS and nSES as time-varying exposures updated over the follow-up and incorporated covariates updated during the follow-up.

We fit Cox proportional hazards regression models, stratified by age and follow-up period and adjusted for covariates, to evaluate whether nDIS or nSES was associated with MM incidence. We report hazard ratios (HRs) and 95% CIs for the incidence of MM by each quartile of nDIS [vs referent, least disadvantaged (Quartile 1)] and nSES [vs referent, most privileged (Quartile 4)]. Simple models were adjusted for age (continuous) as the timescale and cumulative average BMI (<25, 25–29, 30–34, ≥35 kg/m^2^). Multivariable (MV) models were additionally adjusted for cumulative average BMI (<25, 25–29, 30–34, ≥35 kg/m^2^), educational attainment (≤12, 13–15, ≥16 years), alcohol consumption (<1, 1–6, ≥7 drinks/week), cigarette smoking (current, past, never), physical activity (none, <5, ≥5 hours/week), and region of residence (Northeast, South, Midwest, West). Covariates were treated as time-varying in analyses. Separate models were fitted to examine the effect of nDIS or nSES and MM incidence within the strata of education dichotomized at high-school completion (≤12 vs >12 years). We used the missing-indicator method to account for missingness in covariates (generally ≤3%).

Tests for statistical significance were two-sided. *P* for trend was assessed by modeling the time-varying nDIS and nSES scores as continuous variables. Statistical analyses were performed by using SAS statistical software, version 9.4 (SAS Institute, Cary, NC). This study was approved by the Institutional Review Board (IRB) of the Boston University Medical Campus and the IRBs of the participating cancer registries. Return of the baseline and follow-up questionnaires implied informed consent.

## Results

We identified 276 MM cases over 27 years of follow-up, with a median time to diagnosis of 18 years. Age-adjusted baseline characteristics are presented in Table 1, overall and for extreme quartiles of nDIS. Participants who lived in areas with the highest concentrated disadvantage were more likely to be obese (36% vs 22%), smoke cigarettes (21% vs 11%), and reside in the Northeast region (34% vs 24%) of the USA and less likely to have graduated from college (32% vs 58%) and to engage in physical activity (55% vs 65%) compared with those in areas of lowest disadvantage. There was no difference in alcohol consumption between participants living in the most or least disadvantaged neighborhoods (Table 1).

**Table 1.** Age-standardized baseline characteristics of the study population and by top and bottom quartiles of nDIS.

Characteristic	(*n* = 52 272)	Quartile 1:least disadvantaged	Quartile 4:most disadvantaged
Age (years) [mean (SD)]^a^	38.8 (10.7)	39.0 (10.2)	39.0 (11.2)
BMI (kg/m^2^) (%)*			
<25	37	44	31
25–29	31	31	30
30–34	16	13	19
≥35	13	9	17
Cigarette smoking (%)			
Current	16	11	21
Past	20	20	20
Never	65	68	59
Alcohol consumed (drinks/week) (%)			
<1	70	70	69
1–6	22	23	22
≥7	7	6	8
Educational attainment (years)			
≤12	17	10	26
13–15	36	31	41
≥16	46	58	32
Physical activity (hours/week) (%)			
None	40	36	44
<5	52	55	49
≥5	8	10	6
Region of residence (%)			
Northeast	28	24	34
South	30	30	22
Midwest	24	20	34
West	19	26	10

a Value is not age-adjusted.

* Total may not sum to 100% due to missingness (≤3%).

Women living in areas of concentrated disadvantage had a 51% increase in the incidence of MM (Quartile 4 vs Quartile 1, HR: 1.51, 95% CI: 1.06, 2.15, *P* trend: .002 and HR: 1.48, 95% CI: 1.02, 2.13, *P* trend: .003) in age-adjusted and MV-adjusted analyses, respectively (the change in estimates was primarily affected by the inclusion of BMI in the MV models). We also observed a 35% higher incidence of MM among women living in areas of low nSES compared with those in areas of high nSES (HR: 1.35, 95% CI: 0.93, 1.96, *P* trend: .14). Again, the results were similar in fully adjusted models (HR: 1.28, 95% CI: 0.87, 1.90, *P* trend: .31) (Table 2).

**Table 2.** HRs and 95% CIs for the association between neighborhood factors and MM risk.

**Exposures**	**Cases**	**Person-years**	**Age-adjustedHR (95% CI)** ^a^	**MV-adjusted*R* (95% CI)** ^b^
**nDIS**				
Quartile 1 (lower)	52	291 621	1.00 (ref.)	1.00 (ref.)
Quartile 2	56	286 070	1.13 (0.78, 1.65)	1.10 (0.76, 1.61)
Quartile 3	67	281 167	1.29 (0.90, 1.85)	1.25 (0.87, 1.80)
Quartile 4 (higher)	79	269 082	1.51 (1.06, 2.15)	1.48 (1.02, 2.13)
*P* trend^c^			.002	.003
**nSES**				
Quartile 1 (lower)	67	268 096	1.35 (0.93, 1.96)	1.28 (0.87, 1.90)
Quartile 2	63	278 658	1.28 (0.87, 1.86)	1.22 (0.83, 1.79)
Quartile 3	57	286 063	1.19 (0.81, 1.75)	1.15 (0.78, 1.69)
Quartile 4 (higher)	48	289 767	1.00 (ref)	1.00 (ref)
*P* trend^c^			.14	.31

a HRs adjusted for age (continuous) and time period (categorical).

b HRs additionally adjusted for cumulative average BMI (<25, 25–29, 30–34, ≥35 kg/m^2^), cigarette smoking (current, past, never), alcohol consumption(<1, 1–6, ≥7 drinks/week), educational attainment (≤12, 13–15, ≥16 years), physical activity (none, <5, ≥5 hours/week), and region of residence (Northeast, South, Midwest, West).

c Continuous *P* trend.

The results stratified by educational attainment are presented in Table 3. Positive associations of neighborhood disadvantage and MM risk were apparent among women with ≤12 years of formal education and those with at least some college: age-adjusted HR was 1.87 (95% CI: 1.00, 3.48) and slightly attenuated in MV-adjusted models (HR: 1.79, 95% CI: 0.95, 3.37) for high vs low nDIS, respectively. HRs in the strata of completing some college were 1.20 in both the age-adjusted and MV-adjusted models. Similar patterns were observed in nSES among women who completed at least some college (age-adjusted HR for low vs high nSES was 1.23, 95% CI: 0.92, 1.65, and MV-adjusted HR was 1.21, 95% CI: 0.90, 1.63). No association was observed among women with ≤12 years of formal education (Table 3).

**Table 3.** HRs and 95% CIs for the association between neighborhood-level factors and MM risk by educational attainment.

	**≤12 years of education**				**>12 years of education**			
**Exposures**	**Cases**	**Person-years**	**Age-adjustedHR (95% CI)** ^a^	**MV-adjustedHR (95% CI)** ^b^	**Cases**	**Person-years**	**Age-adjustedHR (95% CI)** ^a^	**MV-adjustedHR (95% CI)** ^b^
**nDIS**								
Quartiles 1 and 2 (lower)	13	65 010	1.00 (ref.)	1.00 (ref.)	95	510 833	1.00 (ref.)	1.00 (ref.)
Quartiles 3 and 4 (high)	43	111 100	1.87 (1.00, 3.48)	1.79 (0.95, 3.37)	103	437 057	1.20 (0.91, 1.59)	1.20 (0.90, 1.59)
**nSES**								
Quartiles 1 and 2 (low)	35	116 160	1.06 (0.59, 1.89)	0.97 (0.53, 1.78)	95	428 564	1.23 (0.92, 1.65)	1.21 (0.90, 1.63)
Quartiles 3 and 4 (higher)	17	57 282	1.00 (ref)	1.00 (ref)	88	516 620	1.00 (ref)	1.00 (ref)

a HRs adjusted for age (continuous) and time period (categorical).

b HRs additionally adjusted for cumulative average BMI (<25, 25–29, 30–34, ≥35 kg/m^2^), cigarette smoking (current, past, never), alcohol consumption(<1, 1–6, ≥7 drinks/week), physical activity (none, <5, ≥5 hours/week), and region of residence (Northeast, South, Midwest, West).

## Discussion

In this large, prospective cohort of Black women living in the USA, we found that women living in areas of concentrated disadvantage were at a higher risk of MM regardless of educational attainment. Specifically, Black women who lived in the most disadvantaged neighborhoods had a nearly 50% increased risk of MM in age-adjusted models, and after controlling for well-established confounders such as BMI, compared with those who lived in the least disadvantaged (most privileged) neighborhoods. We observed positive, yet not statistically significant, associations for nSES and MM incidence.

While residence in disadvantaged neighborhoods has been evaluated in relation to several cancer types, to the best of our knowledge, no prior studies have examined the association with MM. Prior studies have shown that low SES, primarily Black neighborhoods, had lower walkability scores [36] and a review by Adkins *et al.* highlights the walkability constraints of neighborhood residence for disadvantaged groups [37]. Two studies that evaluated walkability in relation to MM incidence reported an inverse association [25, 26]. Although we measured different constructs of neighborhood context, our findings of a positive association between living in areas of concentrated disadvantage or low SES and MM incidence are consistent with those of these prior reports.

Several studies have investigated the role of individual SES with MM incidence with measures of association by comparing low to high SES ranging from 1.10 to 1.91 [27–31]. In the present study, we observed an increased risk of MM associated with residence in low-SES neighborhoods after accounting for individual SES (educational attainment) and found that participants were still at an increased risk of MM (average 28% increased risk across nSES models). Our inclusion of neighborhood-level measures provides a deeper understanding of the association between living in low-SES neighborhoods and MM incidence, offering insights that individual-level factors alone may not capture.

The biological underpinnings for the association of where people live with MM incidence are hypothesized to be rooted in stress and inflammation pathways. Chronic inflammation and inflammatory diseases have been linked to MM incidence [38, 39]. Neighborhoods characterized by concentrated disadvantage and low SES are sources of chronic stress, given the limited resources, high crime rates and perceived threats to safety, limited transportation available, crowding, and noise exposure [40, 41]. Stress activates the hypothalamic–pituitary–adrenal axis and releases cortisol [42]. Chronic cortisol activation leads to the dysregulation of lipid accumulation in adipocytes and increased adipose retention [43]. Neighborhood stressors that are associated with obesity contribute to a higher allostatic load and collectively result in negative health outcomes [44–46], including mortality from MM [47]. Other mechanisms relating to living in areas of disadvantage and low SES may include exposure to higher levels of air pollution [48], limited access to healthy foods, or an unsuitable environment for physical activity [49]. Residence in neighborhoods of disadvantage is more common for Black women compared with White women due to the legacy of racism and residential segregation in the USA, regardless of their individual education and income levels [50]. Therefore, Black women are disproportionately exposed to the stress- and environmental-related impacts of living in disadvantaged neighborhoods, including higher levels of air pollution [48] and psychosocial stress [17]. Thus, it is plausible that neighborhood socioeconomic context could play a role in the observed racial disparities in MM incidence.

Therefore, intentional reinvestment that directly supports the residents of these communities is required to improve the health outcomes associated with living in areas of disadvantage and low SES.

There are a few potential limitations in our study worth discussing. As MM is rare in the BWHS cohort, statistical power was somewhat limited, especially in detecting more modest effect sizes and in stratified analyses. We controlled for several potential confounders; however, we did not have any information on family history of MM. In addition, we did not have any information on duration of residence at baseline and therefore could not evaluate potential latency effects. This study had several strengths worth highlighting as well. First, this is a large, prospective cohort study of Black women in the USA who have been followed since 1995, providing detailed, longitudinal data on residence, allowing time-varying exposure data. Our neighborhood indices are based on a population of 59 000 Black women across the USA, ensuring their relevance to our study population. Additionally, the neighborhood indices were measured at the block-group level, providing more precision in the measures as opposed to indices based on larger-scale geographic units such as census tracts or states.

Future studies to further tease apart specific neighborhood-level factors (e.g. walkability, chronic stress, environmental pollution, opportunities to engage in healthy behaviors) that might influence the risk of MM would be beneficial [25, 26, 51, 52]. A better understanding of the drivers of risk would allow targeted interventions to take place in disadvantaged communities to aid efforts to reduce the MM burden. It may be prudent to also consider other measures of the built environment, social vulnerability, or ethnic enclaves to better understand at-risk communities and to target intervention efforts.

## Conclusion

In conclusion, in this prospective cohort study, we found that women living in areas of concentrated disadvantage were at an increased risk of MM, regardless of educational attainment. These findings were observed in measures of both nDIS and nSES. Ultimately, meaningful and intentional community reinvestment may offer an opportunity to reduce the increasing burden of MM among Black women.

## Ethics approval

This study was approved by the Boston University Medical Campus IRB and the IRBs of the participating cancer registries.

## Data Availability

The data underlying this article cannot be shared publicly due to participants’ informed consent, but are available upon reasonable request from the BWHS contact principal investigator (jpalmer@bu.edu) and https://www.bu.edu/bwhs/for-researchers/ (for researchers).
